# Conference Calendar

**DOI:** 10.1002/cfg.438

**Published:** 2004-12


					Comparative and Functional Genomics
Comp Funct Genom 2004; 5: 655-657.
Published online in Wiley InterScience (www.interscience.wiley.com). DOI: 10.1002/cfg.438
Conference Calendar
March -- May 2005
Animal models
BSAS -- Annual Meeting, April 4-6
http://www.bsas.org.uk/meetings/annual.htm
Bioinformatics
IBC -- InfoTechPharma, March 15-16
http://www.infotechpharma.com/
KEY -- Proteomics and Bioinformatics, April
8-13
http://www.keystonesymposia.org/Meetings/
ViewMeetings.cfm?MeetingID=734
9th Annual Research in Computational
Molecular Biology (RECOMB 2005), May
14-18
http://www.broad.mit.edu/recomb2005/
BioIT World, May 17-19
http://www.bioitworldexpo.com/live/26/
IASTED -- Modelling and Simulation, May
18-20
http://www.iasted.org/conferences/2005/
cancun/ms.htm
CHI -- Cheminformatics, May 24-25
http://www.worldpharmacongress.com/
Functional genomics
CHI -- RNAi for Pathway Analysis, March
22-23
http://www.healthtech.com/2005/rpa/index.asp
EuroSciCon -- RNA Mediated Interference in
Practice, April 8
http://www.regonline.co.uk/eventinfo.asp?
EventId=16245
CHI -- Pathway Analysis for Target and
Compound Evaluation, April 20-22
http://www.chimolecularmed.com/05gdd.asp
RNAi-2005: Chemical Biology to Bio-Drug &
Therapeutic Development, May 1-3
http://www.expressgenes.com/RNAi-2005-main.
htm
EuroSciCon: Identifying Gene Expression in
Mammalian Development and Disease, May 20
http://www.regonline.co.uk/eventinfo.asp?
EventId=16304
Human Genomics and Genetics
ICGEB/ESF-FG -- The pathology of pre-mRNA
splicing, April 7-9
http://www.icgeb.org/MEETINGS/CRS05/
ESF.htm
HUGO -- Human Genome Meeting
(HGM2005), 18-21 April
http://hgm2005.hgu.mrc.ac.uk/
ESHG -- European Human Genetics
Conference, May 7-10
http://www.eshg.org/ehgc.htm
Mutation Detection 2005: VIII Intl. Symp. on
Mutations in the Genome, May 31-June 4
http://www.genomic.unimelb.edu.au/santorini.
html
General
Experimental Biology, April 2-6
http://www.faseb.org/meetings/eb2005/call/
default.htm
Copyright  2005 John Wiley & Sons, Ltd.
656 Conference Calendar
ASBMB -- 2005 Annual Meeting, April 2-6
http://asbmb.interliant.com/asbmb/site.nsf/
Sub/2005AnnualMeeting
CHI -- Genomic and Proteomic Sample
Preparation, May 4-5
http://www.healthtech.com/2005/smp/index.asp
Bio Nano Conference, May 8-12
http://www.nsti.org/BioNano2005/
CSHL -- The Biology of Genomes, May 11-15
http://meet1.cshl.edu/meetings/genome05.shtml
Microbes
SGM -- 156th Meeting, April 4-7
http://www.sgm.ac.uk/meetings/MTGPAGES/
hw.cfm
TIGR -- Genomes 2005: April 13-16
http://www.tigr.org/conf/
ASM -- Beneficial Microbes, April 17-21
http://www.asm.org/Meetings/index.asp?
bid=27608
Pharmacogenomics/genomics and
disease
IBC -- Biomarker Pipeline: From Discovery to
Clinical Trials, March 14-16
http://www.ibclifesciences.com/biomarkers
IBC -- Drug Discovery Technology Europe,
March 14-17
http://www.drugdisc.com/europe/
CHI -- Glycomics and Carbohydrates in Drug
Development, March 21-22
http://www.healthtech.com/2005/gly/index.asp
CHI -- Molecular Diagnostics, April 20-22
http://www.chimolecularmed.com/05mdx.asp
CHI -- Microarrays in Medicine, May 4-5
http://www.healthtech.com/2005/mar/index.asp
EuroMedLab 2005, May 8-12
http://www.glasgow2005.org/index.html
CHI -- The World Pharmaceutical Congress,
May 24-26
http://www.worldpharmacongress.com/
CHI -- The Biomarker World Congress, May
23-25
http://www.healthtech.com/2005/bmc/index.asp
Plants
47th Maize Genetics Conference, March 10-13
http://www.maizegdb.org/maize meeting/2005/
SEB -- Plant Frontier Meeting, March 21-23
http://www.sebiology.org/Animal/pageview.asp?
S=2&mid83
Proteomics
KEY -- Proteomics and Bioinformatics, April
8-13
http://www.keystonesymposia.org/Meetings/
ViewMeetings.cfm?MeetingID=734
VIth European Symposium of The Protein
Society, April 30-May 4
http://www.proteinsociety2005.info/barcelona.
html
CHI -- Difficult to Express Proteins, May
16-17
http://www.healthtech.com/2005/dpx/index.asp
CHI -- Phage Display for Engineering Protein
Therapeutics, May 16-17
http://www.healthtech.com/2005/pgd/index.asp
Systems biology
CSHL -- Systems Biology: Global Regulation of
Gene Expression, March 17-20
http://meet1.cshl.edu/meetings/systems05.shtml
Copyright  2005 John Wiley & Sons, Ltd. Comp Funct Genom 2004; 5: 655-657.
Conference Calendar 657
KEY -- Systems and Biology, April 8-13
http://www.keystonesymposia.org/Meetings/
ViewMeetings.cfm?MeetingID=733
Transcriptomics/regulation
CHI -- Quantitative PCR, March 21-22
http://www.healthtech.com/2005/qpc/index.asp
KEY -- chromatin modification pathways,
March 31-April 5
http://www.keystonesymposia.org/Meetings/
ViewMeetings.cfm?MeetingID=745
BS -- Transcription UK, April 13-15
http://www.biochemistry.org/meetings/
programme.cfm?Meeting No=SA035
EMBO -- Chromatin and Epigenetics, May
19-22
http://www-db.embl-heidelberg.de/jss/servlet/
de.embl.bk.embo.EmboSeminarlist#11
Biotech industry
Biomedex 2005: Canadian Biotechnology
Conference, March 30-31
http://www.biomedex.info/
IBC -- European BioProcess International,
April 12-13
http://www.ibc-lifesci.com/bpi/
ACS/BIO/NABC -- World Congress on
Industrial Biotechnology and Bioprocessing,
April 20-22
http://www.bio.org/worldcongress/
Key
ACS: American Chemical Society:
http://www.acs.org/
ASBMB: American Society for Biochemistry and
Molecular Biology: http://www.asbmb.org/
ASM: American Society for Microbiology:
http://www.asm.org/
BIO: Biotechnology Industry Organization:
http://www.bio.org/
BS: Biochemical Society:
http://www.biochemistry.org/
BSAS: British Society of Animal Science:
http://www.bsas.org.uk
CHI: Cambridge Healthtech Institute:
http://www.healthtech.com
CSHL: Cold Spring Harbor Laboratories:
http://meetings.cshl.org/index.htm
EMBO: European Molecular Biology Organisa-
tion: http://www.embo.org/
ESF-FG: European Science Foundation Programme
in Functional Genomics:
http://www.functionalgenomics.org.uk/
ESHG: European Society of Human Genetics:
http://www.eshg.org/
EuroSciCon: http://www.euroscicon.com/
HUGO: Human Genome Organisation:
http://www.gene.ucl.ac.uk/hugo/
IASTED: International Association of Science and
Technology for Development:
http://www.iasted.org/
IBC: IBC Conferences: http://www.ibc-lifesci.com
ICGEB: International Centre for Genetic Engi-
neering and Biotechnology: http://www.icgeb.org/
index.htm
KEY: Keystone Symposia:
http://www.keystonesymposia.org/
NABC: National Agricultural Biotechnology Coun-
cil: http://nabc.cals.cornell.edu/
SEB: Society for Experimental Biology:
http://www.sebiology.org/
SGM: Society for General Microbiology:
http://www.sgm.ac.uk/
TIGR: The Institute for Genomic Research:
http://www.tigr.org/
The Conference Calendar is a listing of forthcoming conferences of interest to our readership. We provide
the title, dates and web address of each conference, to enable readers to find out more information.
Inclusion does not imply endorsement by the journal. If you know of a relevant conference, please
contact the Managing Editor.
Copyright  2005 John Wiley & Sons, Ltd. Comp Funct Genom 2004; 5: 655-657.

				

